# Preliminary Evaluation of Iron Oxide Nanoparticles Radiolabeled with ^68^Ga and ^177^Lu as Potential Theranostic Agents

**DOI:** 10.3390/nano12142490

**Published:** 2022-07-20

**Authors:** Evangelia-Alexandra Salvanou, Argiris Kolokithas-Ntoukas, Christos Liolios, Stavros Xanthopoulos, Maria Paravatou-Petsotas, Charalampos Tsoukalas, Konstantinos Avgoustakis, Penelope Bouziotis

**Affiliations:** 1 Institute of Nuclear & Radiological Sciences & Technology, Energy & Safety, National Center for Scientific Research “Demokritos”, 15341 Athens, Greece; salvanou@rrp.demokritos.gr (E.-A.S.); liolios.xr@gmail.com (C.L.); staxan@rrp.demokritos.gr (S.X.); mparavatou@rrp.demokritos.gr (M.P.-P.); ctsoukal@rrp.demokritos.gr (C.T.); 2 Department of Pharmacy, School of Health Sciences, University of Patras, 26504 Patras, Greece; kolokithas@upatras.gr (A.K.-N.); avgoust@upatras.gr (K.A.); 3 Laboratory of Medicinal Chemistry, Section of Pharmaceutical Chemistry, Department of Pharmacy, National and Kapodistrian University of Athens, Panepistimiopolis-Zografou, 15771 Athens, Greece

**Keywords:** Gallium-68, Lutetium-177, iron oxide nanoparticles, condensed clusters, MTT, radiolabeling, biodistribution, in vivo tracking

## Abstract

Theranostic radioisotope pairs such as Gallium-68 (^68^Ga) for Positron Emission Tomography (PET) and Lutetium-177 (^177^Lu) for radioisotopic therapy, in conjunction with nanoparticles (NPs), are an emerging field in the treatment of cancer. The present work aims to demonstrate the ability of condensed colloidal nanocrystal clusters (co-CNCs) comprised of iron oxide nanoparticles, coated with alginic acid (MA) and stabilized by a layer of polyethylene glycol (MAPEG) to be directly radiolabeled with ^68^Ga and its therapeutic analog ^177^Lu. ^68^Ga/^177^Lu- MA and MAPEG were investigated for their in vitro stability. The biocompatibility of the non-radiolabeled nanoparticles, as well as the cytotoxicity of MA, MAPEG, and [^177^Lu]Lu-MAPEG were assessed on 4T1 cells. Finally, the ex vivo biodistribution of the ^68^Ga-labeled NPs as well as [^177^Lu]Lu-MAPEG was investigated in normal mice. Radiolabeling with both radioisotopes took place via a simple and direct labelling method without further purification. Hemocompatibility was verified for both NPs, while MTT studies demonstrated the non-cytotoxic profile of the nanocarriers and the dose-dependent toxicity for [^177^Lu]Lu-MAPEG. The radiolabeled nanoparticles mainly accumulated in RES organs. Based on our preliminary results, we conclude that MAPEG could be further investigated as a theranostic agent for PET diagnosis and therapy of cancer.

## 1. Introduction

Nanoparticles (NPs) have been proposed as potential delivery systems for the simultaneous monitoring and therapy of various diseases, including cancer [1,2,3,4]. The key characteristic of nanoparticles is multivalency, a term that describes their ability to carry a multitude of chemotherapeutic agents and probe molecules. More specifically, magnetic iron oxide nanoparticles (MIONs) can be modified with biocompatible polymers for improved bioavailability. Furthermore, their specificity against cancer cells can be increased by adding molecular targeting moieties (antibodies/peptides, etc.) in their structures [5,6,7]. More recently, NPs have been investigated as nanobrachytherapy agents after intratumoral injection [8,9,10,11,12]. Among the various kinds of recently studied modifications of NPs is radiolabeling, which has been suggested for both diagnostic imaging and therapy of cancer [1,13,14,15].

The radiolabeling of nanoparticles usually entails the incorporation of a radioactive atom to the NP either directly or via a chelator [16,17], activation of nanoparticles by neutron irradiation [12,18], encapsulation of radioactive atoms [19], or the formation of nanoparticle vehicles using radioactive atoms [20]. The main issue when approaching the radiolabeling of NPs is the selection of the appropriate radioisotope, which depends on different factors and mainly on the intended application [1,13,14]. For example, gamma rays or positron-emitting radioisotopes are ideally suited for in vivo tracking of NPs. Gamma rays, either directly emitted or generated after the annihilation of positrons, have a high penetration capacity, which enables the external detection of the NP loci by using SPECT (Single-photon emission computed tomography) or PET cameras in a non-invasive way after administration into a living organism [1,13,14,21].

Radiolabeled MIONs have been investigated as multimodal imaging probes utilizing SPECT or PET modalities with Magnetic Resonance Imaging (MRI) since their spin-active iron oxide core can act as the necessary MRI contrast agent component [22,23]. Over the last years MIONs have been labelled with SPECT (Technetium-99m: ^99m^Tc, Thallium-201: ^201^Tl, Indium-111: ^111^In, Iodine-131: ^131^I, Iodine-123: ^123^I, Gallium-67: ^67^Ga) [1,13,24,25] and PET (Fluorine-18: ^18^F, Carbon-11: ^11^C, Nitrogen-13: ^13^N, Oxygen-15: ^15^O, Iodine-124: ^124^I, Copper-64: ^64^Cu, Gallium-68: ^68^Ga) radioisotopes [4,17,26,27,28,29,30]. Although multimodal PET/Computed Tomography (CT) imaging is now a well-established routine in Nuclear Medicine [31,32], it has certain shortcomings, such as the inability to perform simultaneous data acquisition, as well as the significant radiation dose received by the patient during CT [13]. On the other hand, MRI offers better contrast for soft tissues as well as functional-imaging capabilities. Therefore, hybrid imaging with PET/MR has recently gained favor in preclinical research as a very promising tool that will soon progress to routine clinical application [26,31,33]. In this regard, of particular interest are magnetic nanoparticles (MNPs) labeled with ^68^Ga, (t_1/2_ = 1.14 h, β^+^ 90%, EC 10%, 770 keV, 1880 keV), a PET radioisotope which can be easily produced on-site from ^68^Ge/^68^Ga generators. ^68^Ga is similar to the successfully used ^99^Mo/^99m^Tc generator system in ^99m^Tc-radiopharmacy and also presents the option of labeling with cold kit formulations [34]. Moreover, due to the inherent magnetic properties of MIONs, the final product, ^68^Ga-MIONs, could be utilized for PET/MR dual-modality imaging [27,28].

Regarding NPs, which are intended for radiotherapy applications, the option of choice is a particle-emitting radioisotope with high linear energy transfer (LET in keV/um), which reflects energy deposition and, therefore, ionization density along the track of a charged particle. Actinium-225 (^225^Ac), which is an alpha- (α) emitter, and Yttrium-90 (^90^Y) and Lutetium-177 (^177^Lu), which are beta- (β) emitters, are such radioisotopes. [1,9,14,21,35,36,37,38,39,40]. ^177^Lu has emerged as a pivotal radioisotope due to its suitable nuclear decay characteristics. It has numerous advantages when compared to other therapeutic radioisotopes (Gold-198: ^198^Au, Yttrium-90: ^90^Y, Phosphorus-32: ^32^P, Rhenium-186: ^186^Re, etc.), such as the emission of β−particles, their energies and abundance (t_1/2_ = 6.71 d, E_β[max]_ = 497 keV, E_γ_ = 113 keV [6.4%], 208 keV [11%] and 0.7 mm range in tissue) [41]. Beta-particles are negatively charged electrons with low LET energy (≈ 0.2 keV/um). They are usually regarded as more efficient for the treatment of solid, heterogeneous, or large-volume tumors since the long range of these emitted electrons leads to the crossfire phenomenon, which affects all the cells found within range of the decaying atoms, thus minimizing the need to target every cell within the tumor [35]. However, their therapeutic efficacy predicates the presence of very high radioisotope concentrations within targeted tissue; therefore, the facile and efficient radiolabeling of the delivery systems is of the utmost importance.

Previous studies mention the development of iron oxide nanoparticles with a condensed magnetic core, decorated with different polymers and, in some cases, with targeting moieties [42,43,44,45,46]. The condensed clustering structure refers to the in situ clustering of MIONs during crystal growth, in such a fashion that the individual MIONs adopt the same crystallographic orientation with their neighboring crystals through epitaxial aggregation [42,47,48]. This dense packing of MIONs can dramatically enhance the magnetic properties of the synthesized NPs, boosting their performance in magnetic targeting, magnetic hyperthermia, photothermal therapy, and MRI applications [46,49,50,51]. Such attributes make MIONs of co-CNCs a highly attractive platform for further derivatization with radioisotopes towards combinatorial approaches to tackle cancer and incorporate multimodal imaging techniques to a single theranostic agent.

In the present work, we have focused on the development of a theranostic agent based on MIONs of co-CNCs, coated with alginic acid (MA) and stabilized by a layer of polyethylene glycol (MAPEG) for efficient radiolabeling with a diagnostic and a therapeutic isotope. The co-CNCs herein were synthesized through a soft biomineralization route at 50 °C and ambient pressure, as previously reported [42], and then labeled with ^68^Ga and ^177^Lu without the use of chelators via the rich-in-carboxylates alginate coating. Radiolabeling efficiency was determined with instant thin-layer chromatography (ITLC-SG), and the radiolabeled MNPs were also investigated with the same system over time to assess their stability. The basic physicochemical characteristics of the radiolabeled assemblies were determined by dynamic light scattering (DLS), and their compatibility with red blood cells (RBCs) as well as their in vitro cytocompatibility in 4T1 cells (murine mammary carcinoma), were investigated. Finally, their biodistribution profile at predetermined time points was investigated ex vivo in normal mice.

## 2. Materials and Methods

*Warning! ^68^Ga and ^177^Lu isotopes present serious health threats and require special radioprotective precautions during handling to reduce the risk of harm. All radiolabeling procedures and work associated with radiolabeled compounds was conducted in a radiochemistry facility which has all the necessary infrastructure, expertise, and licensing to safely conduct experiments with radioisotopes.*

The buffer used for radiolabeling was prepared from trace-free reagents (Sigma-Aldrich, Munich, Germany). The 4T1 murine mammary carcinoma cell line was acquired from the cell bank of the Laboratory of Radiobiology, Institute of Nuclear & Radiological Sciences & Technology, Energy & Safety, NCSR “Demokritos”. The cells were free of mycoplasma contamination, as judged visually under microscope observation and by regular 4′,6-diamidine-2′-phenylindole dihydro-chloride (DAPI) staining of the cell cultures. The media for the cultures were purchased from Biowest (Riverside, MO, USA), and the MTT reagent (3-[4,5-dimethylthiazol-2-yl]-2,5-diphenyl-tetrazolium bromide) was obtained from Applichem (Darmstad, Germany). Optical density measurements in the in vitro experiments were conducted using a LabSystems Multiskan RC Microplate Reader (Thermo Fisher Scientific, MA, USA). A lower-activity commercial Ge-68/Ga-68 generator was acquired from Eckert & Ziegler (Berlin, Germany). Lutetium-177 was purchased from POLATOM (Otwock, Poland). Water for injection was purchased from DEMO S.A. (Krioneri Attiki, Greece). All other reagents and solvents used in these studies were obtained from commercial sources without further purification. The radioactivity of [^68^Ga]GaCl_3_, [^177^Lu]LuCl_3_, and the radiolabeled nanoparticles was measured using a dose calibrator (Capintec, Ramsey, NJ, USA). Glass microfiber chromatography paper impregnated with silica gel (iTLC-SG) were purchased from Agilent Technologies (Santa Clara, CA, USA) and along with a Radio-TLC Scanner (Scan-Ram, LabLogic, Sheffield, UK) were used in the determination of radiolabeling yield/purity during radiolabeling and stability studies. Water was deionized to 18 MΩ⋅cm using an easy-pure water filtration system (Barnstead International, Dubuque, IA, USA). A gamma scintillation counter, Cobra II (Canberra, Packard, Downers Grove, IL, USA), was used to measure the radioactivity of each organ and blood sample in ex vivo biodistribution studies.

For the animal experiments, female CFW mice were used. The animals were housed in air-conditioned rooms under a 12-h light/dark cycle and allowed free access to food and water. The animals were obtained from the breeding facilities of the Institute of Biosciences and Applications, NCSR “Demokritos”. Our experimental animal facility is registered according to the Greek Presidential Decree 56/2013 (Reg. Number: EL 25 BIO 022), in accordance with the European Directive 2010/63, which is harmonized with national legislation regarding the protection of animals used for scientific purposes. All applicable national guidelines for the care and use of animals were followed. The study protocol was approved by the Department of Agriculture and Veterinary Service of the Prefecture of Athens (Protocol Number: 1606/11-04-2018). These studies have been further approved by our institutional ethics committee, and the procedures followed are in accordance with institutional guidelines.

### 2.1. Synthesis of MIONs

#### 2.1.1. Synthesis of MA and MAPEG

The synthesis of alginate-coated MIONs (namely MA) of co-CNCs and their subsequent PEGylation were performed according to our previously described method [43]. Briefly, the alkaline precipitation of co-CNCs MIONs was performed from a single ferrous precursor of FeSO_4_·7H_2_O in the presence of sodium alginate (NaAlg) from brown algae (typical average weight 67 kDa, 100e300 cps, Sigma) as the first polymeric coating of the magnetic nanoparticles at 50 °C for 80 min. To remove the unbound polymer and byproducts of the reaction, the crude product was centrifuged twice at 16,000 rpm for 40 min. The precipitate was collected and redispersed in an equal volume of water. A third, mild centrifugation, was performed at 2000 rpm for 10 min to remove bigger aggregates of MIONs. The supernatant was collected and stored at 4 °C for further use. For the PEGylation of MIONs (namely MAPEG), coupling reagents such as DIC (*N*,*N’*-diisopropylcarbodiimide, Aldrich), HOBt (hydroxybenzotriazole, CBL, Patras), and DIPEA (*N*,*N*-Diisopropylethylamine, Merck, KGaA, Darmstadt, Germany), were used for the conjugation of MeO-PEG-NH_2_ (average molecular mass 2000, RAPP Polymere GmbH, Tübingen, Germany) in DMF (for peptide synthesis, Acros Organics, Geel, Belgium). Conjugation reagents were used in a molar excess of 3.3 with respect to the carboxylates present on MA, while mPEG-NH_2_ was used in a molar excess of 3. After each reaction step, the nanoparticles were washed twice with DMF through centrifugation (30 min at 16,000 rpm) in order to remove the byproducts of the reaction. After the final reaction step, two washings with DMF were performed, followed by three more washings (30 min at 16,000 rpm) with ultra-pure H_2_O for the preparation of the aqua PEGylated MIONs.

#### 2.1.2. Dynamic Light Scattering

The determination of the hydrodynamic diameter (Dh) and polydispersity index of nanoparticles dispersed in deionized H_2_O was performed with a ZetaSizer Nano series Nano-ZS (Malvern Instruments Ltd., Malvern, UK) equipped with a He–Ne laser beam at a wavelength of 633 nm and a fixed backscattering angle of 173°. The concentration of the colloid suspensions used for the measurement was 0.0125% *w/v* (g/100 mL) in Fe_2_O_3_. The ζ-potential of the nanoparticles was measured with the same instrument as the average of 100 runs with the phase analysis light scattering mode (PALS) after equilibration at 25 °C.

### 2.2. Radiolabeling MIONs with ^68^Ga

Gallium-68 was initially eluted from a ^68^Ge/^68^Ga generator with 7 mL of 0.1 N HCl as [^68^Ga]GaCl_3_ and trapped onto an acidic cation-exchange resin (Bio-Rad AG 50W-X8 cation exchanger < 400 mesh). Metal impurities were removed by an acetone solution (80 *v/v*%) and 0.15 M HCl (20 *v/v*%). The desorption of purified ^68^Ga was accomplished with a solution of acetone (97.6 *v/v*%) and 0.15 M HCl (2.4 *v/v*%) [52]. For the labeling of the nanoparticles, 350 μL of sodium acetate buffer, pH 4, 50 μL of MIONs suspension (C_MA_ = 7.8 mg/mL, C_MAPEG_ = 8.2 mg/mL) and 100 μL of [^68^Ga]GaCl_3_ (10–60 MBq), were incubated at 75 °C for 30 min. The activities of the eluate and labeling mixture were measured with a dose calibrator. The radiochemical yield (RCY) of the labeled MIONs was determined by ITLC-SG, using silica gel sheets as the stationary phase and 0.1 M citric acid as the mobile phase. The [^68^Ga]Ga-NPs remain at the spot of the TLC while unbound ^68^Ga migrates to the solvent front [53]. The radioactivity on the ITLC-SG strips was visualized using a Radio-TLC Scanner. The percentage of radiochemical yield (% RCY) of [^68^Ga]Ga-MIONs was calculated as 100 × (counts at application point/total counts). Data collection and analysis were performed with Laura software v. 5.0.4.29.

### 2.3. Radiolabeling MIONs with ^177^Lu

Lutetium-177 was acquired in the form of [^177^Lu]LuCl_3_ in 0.04 M HCl solution. Direct radiolabeling was achieved for both types of MIONs (MA and MAPEG). Nanoparticles (50 μL, C_MA_ = 7.8 mg/mL, C_MAPEG_ = 8.2 mg/mL) were added to trace-free sodium acetate buffer pH 5.4. Then, 10 to 30 MBq of [^177^Lu]LuCl_3_ were added, after which the mixture was slightly vortexed and consequently incubated at 75 °C for 30 min. For radiochemical analysis, we used ITLC-SG (citric acid, 0.1 M), where [^177^Lu]Lu-MIONs remained at the application point (Rf = 0.0–0.2) while unbound ^177^Lu^3+^ was detected at the solvent front (Rf = 0.8–1.0). The percentage of ^177^Lu incorporated onto the NPs was calculated as 100 × (counts at application point/total counts). Data collection and analysis were performed with Laura software v. 5.0.4.29.

### 2.4. In Vitro Stability Studies of [^68^Ga]Ga-MIONs and [^177^Lu]Lu-MIONs

In order to assess the in vitro stability of the radiolabeled MIONs, samples of the [^68^Ga]Ga-MA and MAPEG, as well as the samples of [^177^Lu]Lu-MA and MAPEG, were incubated with human serum (Sigma–Aldrich) (1:10, *v/v* radiolabeled MIONs: serum) at 37 °C. In the case of the [^68^Ga]Ga-MIONs, serum stability was evaluated by ITLC-SG (citric acid, 0.1 M) at 30, 60, and 120 min post-radiolabeling, whereas aliquots from the [^177^Lu]Lu-MIONs were assessed up to 7 days post-incubation. Bench stability was also assessed for all four radiolabeled nanoconstructs at the same time points as the serum stability assessment. All experiments were performed in triplicate from three independent radiolabeling procedures.

### 2.5. Hemolysis Assay

The biocompatibility of MA and MAPEG with Red Blood Cells (RBCs) was assessed by the hemolysis assay according to previously described protocols [54,55]. The blood samples were centrifuged at 1000× *g* for 5 min to separate the plasma from the RBCs. After removing the plasma, the RBCs were washed 3 times with phosphate buffer saline (PBS, 0.01 M, pH 7.4) free of calcium and magnesium. RBCs (15 μL) were co-incubated for 3 h at 37 °C with samples (500 μL) of different concentrations of MA and MAPEG after serial dilutions with PBS ranging from 4.0625 to 130 μg[Fe_2_O_3_]/mL. Samples with PBS and RBCs without any nanoparticles were used as our negative control (0%). Once RBCs are incubated with water, hemolysis is provoked due to the hypotonic effect of water; thus, this sample was used as our positive control (100%) [56]. At the end of the 3 h incubation, all samples were centrifuged at 1000× *g* for 5 min, and 100 μL of the supernatant was removed and placed in a 96-well plate. The optical density (OD) was measured with a microplate reader at 450 nm. The hemolysis ratio was calculated with the following equation: Hemolysis ratio% = (OD of MIONs−OD of negative control)/(OD of positive control−OD of negative control) × 100.

The experiment was conducted in RBCs isolated from blood samples from healthy donors. All experiments were carried out in accordance with relevant guidelines and regulations and were performed in triplicate. To remove the interfering absorption of the MIONs, a control experiment under the same conditions was conducted, and the absorbance of the supernatant was taken into account. Fluctuations observed in the measurements are in the error range of the instrument.

### 2.6. Cell Cultures

The growth and metastatic pattern of 4T1 cells mimic stage IV human breast cancer. This murine mammary carcinoma cell line was grown in Dulbecco’s modified Eagle’s growth medium (DMEM), pH 7.4, supplemented with 10% FBS, 100 U/mL of penicillin, 2 mM glutamine, and 100 μg/mL of streptomycin. The cell cultures were maintained in flasks and were grown at 37 °C in a humidified atmosphere of 5% CO_2_ in air. Subconfluent cells were detached using a 0.25% trypsin-0.53% mM ethylenediaminetetraacetic acid (EDTA) solution, while the subcultivation ratio was 1:8–1:10.

### 2.7. MTT Toxicity Assay

The in vitro cytotoxicity of the prepared MA and MAPEG MIONs as well as of the [^177^Lu]Lu-MAPEG against 4Τ1 cells was evaluated by the 3-(4,5-dimethylthiazol-2-yl)-2,5-diphenyltetrazolium bromide (MTT) colorimetric assay. Briefly, the cells were seeded in 96-well plates and allowed to grow overnight at 37 °C in a 5% CO_2_ incubator. For the 24 h protocol, 15 × 10^3^ cells were seeded per well, whereas for the 48 and 72 h protocols 8 × 10^3^ and 4 × 10^3^ cells/well were seeded, respectively. The cells were treated with increasing concentrations of both complexes (4.0625, 8.125, 16.25, 32.5, 65, and 130 μg[Fe_2_O_3_]/mL). The ^177^Lu radiolabeled MAPEG was evaluated after 24 h incubation at the same concentrations as the non-radiolabeled counterparts, with the activities ranging from 0.125 to 4 MBq/mL. After the various incubation periods, the medium was removed and replaced with 100 μL of MTT dissolved in the growth medium (1 mg/mL). After 4 h of incubation with the MTT, the latter was aspirated, and isopropanol (100 μL) was used to solubilize the formazan crystals. The absorbance was recorded at 540 nm. All experiments were performed in triplicate, and the results were expressed as noted from the following equation: Cell viability (%) = (mean optical density (OD) of treated cells/mean OD of untreated cells) × 100.

### 2.8. Ex Vivo Biodistribution Studies of the Radiolabeled MIONs

The biological behavior of the radiolabeled MIONs was evaluated in female CFW mice, 6–8 weeks old, weighing 20–30 g (*n* = 3 animals per time-point). According to the experimental protocol, the activity of 100 μL of radiolabeled MIONs suspension was measured in a dose calibrator and administered intravenously via the tail vein (≈20 μg Fe_2_O_3_/100 μL/mouse). Then, at the designated time points, the animals were euthanized in a chamber saturated with isofluorane vapors, and the organs and tissues of interest were excised, weighed, and measured in an automatic γ-counter. All measurements were corrected for background and radioactive decay. Finally, the accumulation of the radiolabeled MIONs in organs and tissues at each time point was expressed as the mean percentage of injected activity per gram ± standard deviation (% IA/g ± SD), using an appropriate sample as a standard. The same concentration of radiolabeled MIONs was administered in the mice for all biodistribution experiments.

For the [^68^Ga]Ga-MA and the [^68^Ga]Ga-MAPEG, 100 μL of the sample with a radioactivity of ≈2.3 ΜBq of a suspension of radiolabeled MIONs in water for injection (1:3) was administered by intravenous injection via the tail vein. At 30-, 60-, and 120-min post-injection, the mice were euthanized by isofluorane inhalation, and the organs, along with blood and muscle samples, were excised and measured.

In the Lutetium group, ≈0.8 ΜBq of [^177^Lu]Lu-MAPEG were diluted as described above and administered intravenously. At 1, 2, and 7 d post-injection, all mice were euthanized, the organs/tissues were excised, and the % injected activity per gram was calculated as mentioned above.

### 2.9. Statistical Analysis

The data are presented as means ± standard deviations (SD). For the MTT and biodistribution studies, data were compared using a two-way ANOVA analysis with a significance level of *p* < 0.05. The asterisks indicate the statistical significance of the difference between the results (* *p* < 0.05, ** *p* < 0.01, *** *p* < 0.001). The absence of asterisks denotes a non-significant statistical difference.

## 3. Results and Discussion

### 3.1. Synthesis and Characterization of MIONs

**Figure 1:**
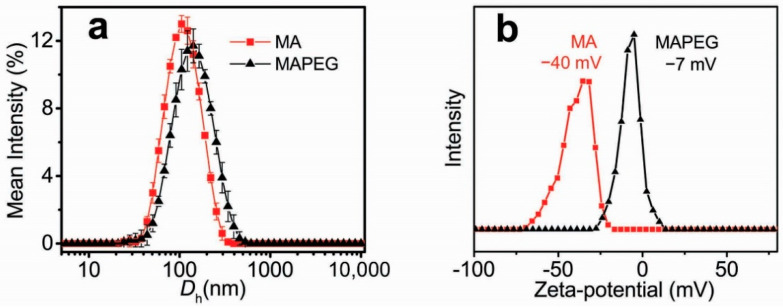
(**a**) Hydrodynamic diameters (*D*_h_’s) and (**b**) ζ-potentials of the plain (MA) and PEGylated (MAPEG) co-CNCs MIONs.

The co-CNCs used in this research were composed of alginate-coated magnetic iron oxide nanocrystallites (MA) and their PEGylated analogs (MAPEG), in order to attribute stealth properties to the nanoparticles. The synthetic pathway followed for the synthesis of these MIONs, as well as their physicochemical and magnetic characterization, has been previously described in detail [42,43]. For the present study, new and freshly synthesized samples were prepared, and their basic physicochemical characterization, concerning their size and zeta-potential, was performed with DLS. As shown in Figure 1, the average size (*D*_h_) of MA nanoparticles was 100 nm with a ζ-potential of −40 mV, owing to the rich-in-carboxylates alginate surface. Their PEGylated counterparts exhibited a *D*_h_ of 120 nm and a significant reduction in ζ-potential to −7 mV due to the conjugated PEG coating.

### 3.2. Radiolabeling of MIONs with ^68^Ga

**Figure 2:**
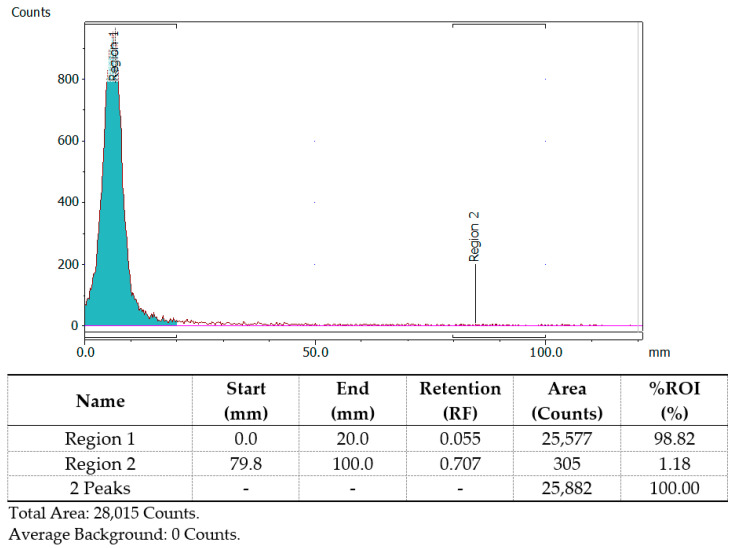
Representative radio-TLC chromatograph of [^68^Ga]Ga-MA or [^68^Ga]Ga-MAPEG obtained after 30 min of incubation at 75 °C.

Radiolabeling with ^68^Ga, as described above, consisted of the incubation of the nanoparticles in the presence of a sodium acetate buffer (pH = 4) and 100μL of [^68^Ga]GaCl_3_ eluate for 30 min at 75 °C. According to the radio-TLC analysis, the radiochemical yields were >90% for both MA and MAPEG after 30 min of incubation at 75 °C (94.28 ± 3.27% for [^68^Ga]Ga-MA and 94.53 ± 2.76% for [^68^Ga]Ga-MAPEG). Fluctuations of the reaction temperature or incubation period had practically no improvement on radiolabeling yield and in vitro stability of the radiolabeled sample. A representative chromatograph of the evaluation of the [^68^Ga]Ga-MIONs with radio-TLC is shown in Figure 2. Overall, the radiolabeling procedures applied in the case of ^68^Ga resulted in highly efficient radiolabeling of both groups, without the need for further purification procedures and without long incubation periods, parameters of vital importance when using radioisotopes with short half-lives. The pH plays an important role during radiolabeling with ^68^Ga, and a range of 3–5 is considered as the most suitable because, in aqueous solutions, ^68^Ga is found primarily in the oxidative state +3 and can bind to electron donors, whereas at high pH values insoluble gallium hydroxides are formed [57].

**Figure 3:**
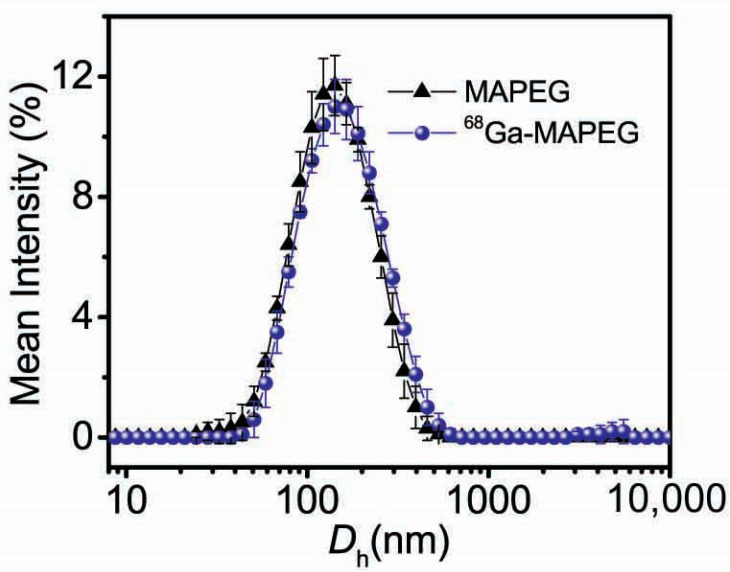
Size distribution of MIONs before (MAPEG) and after (^68^Ga-MAPEG) radiolabeling with ^68^Ga.

In our study, the labeling of MIONs was direct through the binding of the available carboxylic acid groups of the alginic acid with the radioisotope. As shown in Figure 3, the PEGylated nanoparticles preserved their colloidal characteristics after ^68^Ga radiolabeling, exhibiting a *D*_h_ of ≈140 nm and recording only a slight increase of 20 nm.

On the other hand, the plain MA nanoparticles displayed extensive aggregation after the radiolabeling process (*D*_h_ ≈ 600 nm), slowly forming a turbid solution, and finally precipitating after 24 h. This behavior is due to the pKa of the alginate, which is ≈3.5–4.6 [58]. Therefore, the acidic conditions employed for the radiolabeling induced the protonation of the alginate and the loss of electrostatic stabilization of the non-PEGylated MA MIONs.

### 3.3. In Vitro Stability of [^68^Ga]Ga-MIONs

**Figure 4:**
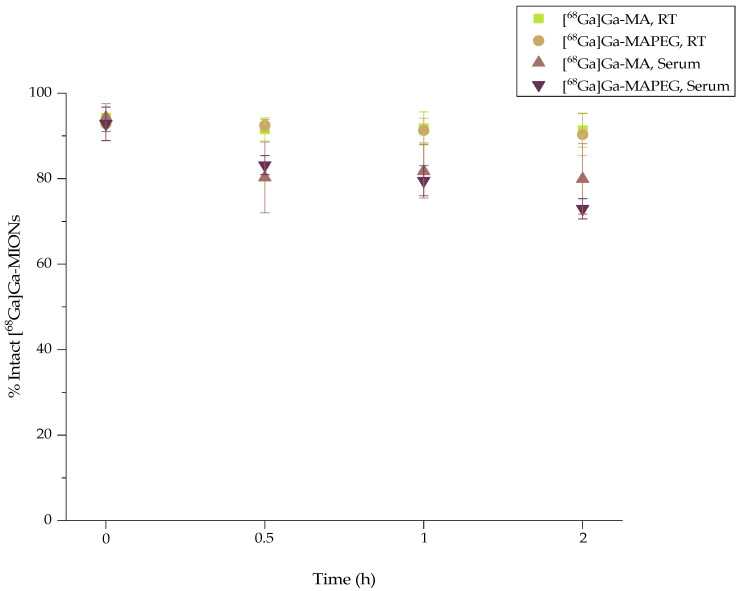
Radiochemical stability of [^68^Ga]Ga-MA and [^68^Ga]Ga-MAPEG at RT and in human serum (x axis not in scale).

Stable binding of the radioisotope onto the nanosystem is of the utmost importance to assure that after administration, background noise due to freely circulating radioisotope during imaging will be minimal. Although in vitro stability does not always ensure in vivo stability, it gives a good indication of the in vivo fate of the radiolabeled compound. Therefore, the stability of the radiolabeled sample at room temperature (RT), as well as its stability in the presence of human serum, were tested before assessing the biological behavior in an animal model (Figure 4). The ITLC-SG analysis at 2 h post-radiolabeling showed that both [^68^Ga]Ga-MA and [^68^Ga]Ga-MAPEG remained intact at room temperature (91.35 ± 3.98% and 90.3 ± 4.92%, respectively). When incubated in human serum (1:10 *v/v*, at 37 °C, 2 h), they also showed moderate stability, i.e., 79.94 ± 8.23% for [^68^Ga]Ga-MIONs, and 72.94 ± 2.38%, for the [^68^Ga]Ga-MAPEG.

### 3.4. Radiolabeling of MIONs with ^177^Lu

**Figure 5:**
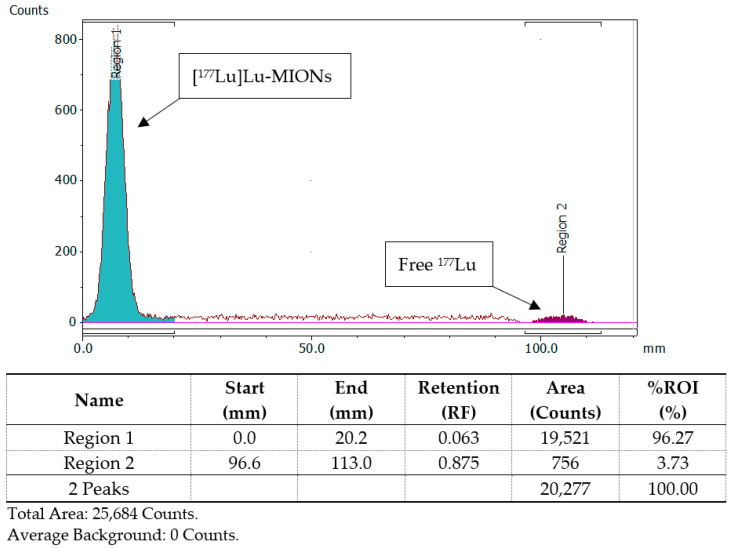
Representative radio-TLC chromatograph of [^177^Lu]Lu-MA or [^177^Lu]Lu-MAPEG after 30 min of incubation at 75 °C.

The ^177^Lu labeling protocol of MA and MAPEG was similar to the one described for the [^68^Ga]Ga-counterparts, where radiolabeling was achieved after the incubation of nanoparticles with [^177^Lu]LuCl_3_ (50 μL, 10–30 MBq) in sodium acetate buffer in a slightly higher pH (pH = 5.4) at the same temperature (75 °C). Radiochemical analysis (ITLC-SG) showed an RCY of 95.21 ± 1.28% and 93.65 ± 1.03% for [^177^Lu]Lu-MA and [^177^Lu]Lu-MAPEG, respectively, after 30 min at 75 °C (Figure 5). These results could be attributed to the stable binding of the positively charged radioisotope Lu^3+^, same as in the case of ^68^Ga^3+^, to the alginate corona and specifically to its negatively charged –COO^−^.

**Figure 6:**
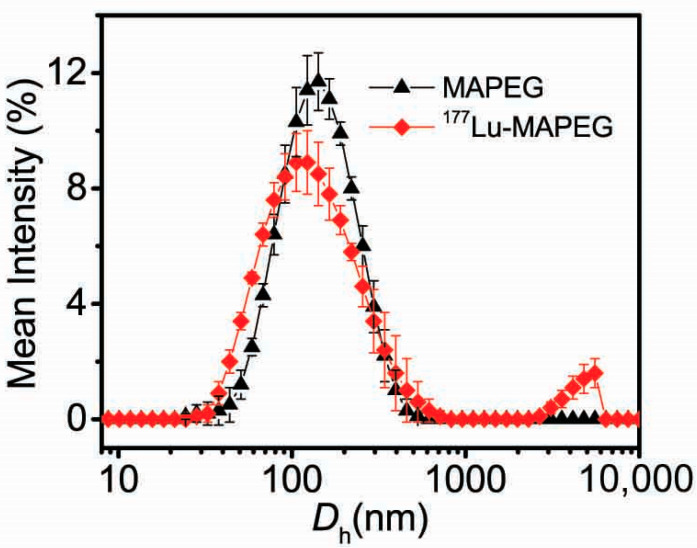
Size distribution of MIONs before (MAPEG) and after (^177^Lu-MAPEG) radiolabeling with ^177^Lu.

The *D*_h_ of the [^177^Lu]Lu-MAPEG was identical (≈119 nm) to the parent MAPEG nanoparticles with no signs of aggregation (Figure 6). The less acidic conditions slightly reduced the aggregation rate of [^177^Lu]Lu-MA nanoparticles, displaying a *D*_h_ ≈ 450 nm, but after 24 h, the nanoparticles had precipitated.

### 3.5. In Vitro Stability of [^177^Lu]Lu-MIONs

**Figure 7:**
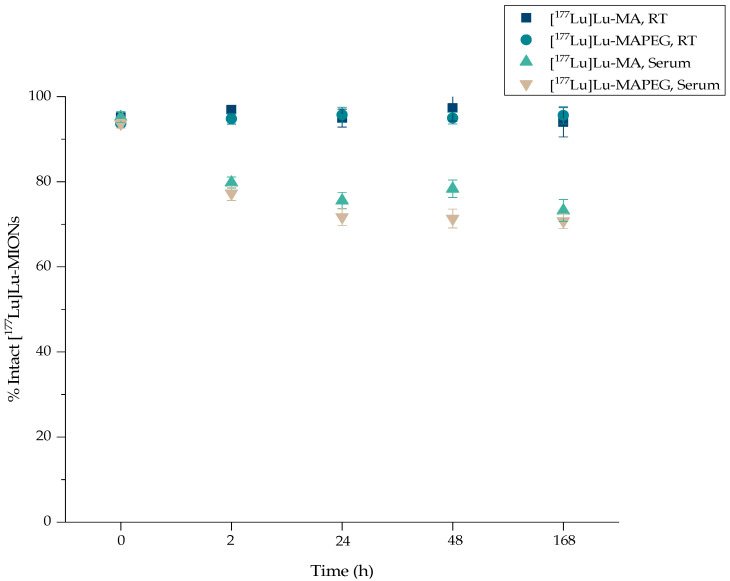
Radiochemical stability of [^177^Lu]Lu-MA and [^177^Lu]Lu-MAPEG at RT and in human serum (x axis not in scale).

The [^177^Lu]Lu-labeled nanoparticles were stable at room temperature up to at least 7 days post-preparation (93.97 ± 3.44% of the [^177^Lu]Lu-MA versus 95.60 ± 2.03% of the [^177^Lu]Lu-MAPEG) (Figure 7). On the other hand, the percentage of intact ^177^Lu-labeled MIONs gradually decreased from 79.81 ± 1.28% after 2 h co-incubation with serum to 73.24 ± 2.59% 7 days later in the case of [^177^Lu]Lu-MA ([^177^Lu]Lu-MA:serum 1:10 *v/v*, at 37 °C). A comparable behavior was demonstrated for the [^177^Lu]Lu-labeled MAPEG ([^177^Lu]Lu-MAPEG:serum 1:10 *v/v*, at 37 °C), where the percentage of intact radiolabeled nanostructures ranged from 77.29 ± 1.69% to 70.72 ± 1.75% at 2 h and 7 d post radiolabeling, respectively. Finally, the ^177^Lu-labeled radiotracer diluted with water for injection (i.e., as used for the biodistribution experiments) was assessed for stability up to 5 days post preparation and was found to remain intact (>90%).

At 2 h post-incubation, serum stability for the ^177^Lu-labeled MIONs was like that shown for the ^68^Ga nanotracers; however, up to 168 h post-incubation, a slightly decreasing trend was observed. A direct comparison with work from other groups cannot be made, as most serum stability studies were performed with a higher NP:serum ratio (1:1 *v/v* or 1:5 *v/v* compared to 1:10 *v/v* in our case). Furthermore, most stability studies ended at 24 or 48 h post-incubation. Finally, labeling with ^177^Lu was accomplished via a chelator, such as 1,4,7,10-Tetraazacyclododecane-1,4,7,10-tetraacetic acid (DOTA), and thus, higher serum stability may be expected [59,60].

### 3.6. Hemolysis Assay

**Figure 8:**
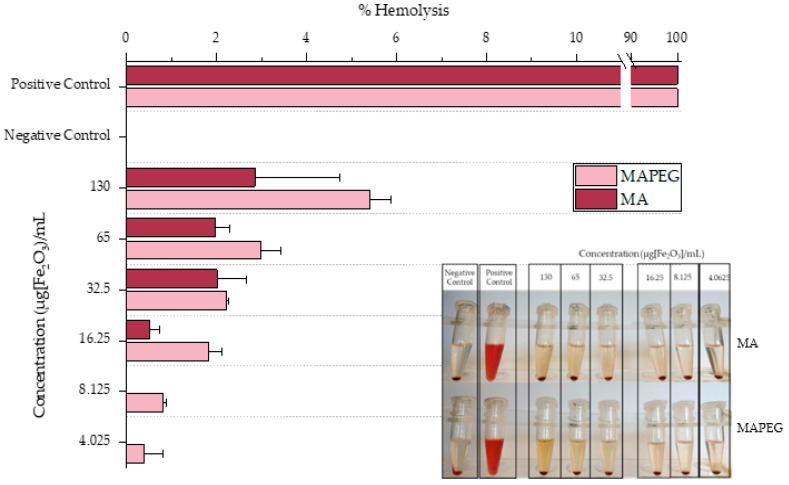
Hemolytic effect of different concentrations of MA and MAPEG. Positive control: 500 μL H_2_O + 15 μL of RBCs; negative control: 500 μL PBS + 15 μL of RBCs.

The hemolytic behaviors of different concentrations of MA and MAPEG against RBCs were investigated and are demonstrated in Figure 8. Low hemolysis levels (<6%) were noted for both nanoconstructs at all concentrations. Even the slightly higher hemolysis ratio indicated by the MAPEG MIONs could be attributed to the more yellowish color of the nanoparticles.

It is of major importance that nanoparticles aiming to serve as theranostic agents have minimal interactions with blood components so as not to compromise their systemic administration. To this end, nanosystems such as iron oxide or gold nanoparticles must prove their biocompatibility before being intravenously injected into a living organism [61,62]. In our case, all samples exhibited minimal hemoglobin release from the RBCs, indicating negligible hemolysis according to the <10% acceptance limit for biopharmaceuticals [63].

### 3.7. In Vitro Toxicity of MIONs

**Figure 9:**
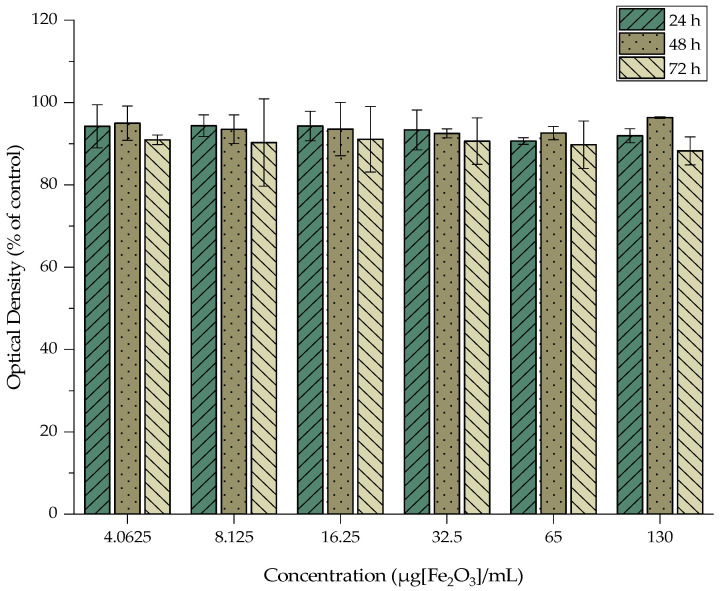
MTT assay of MA against the 4T1 cell line after 24, 48, and 72 h. Mean values (*n* = 3) and the SD (bars) are shown (x axis not in scale).

**Figure 10:**
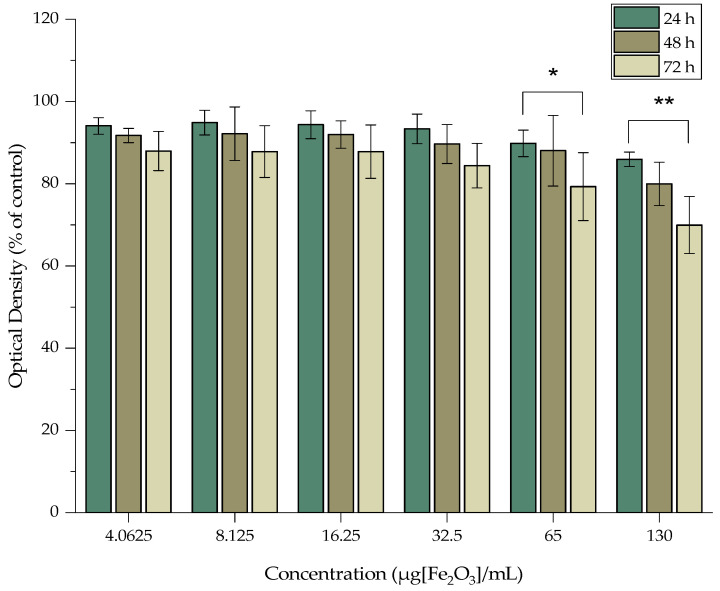
MTT assay of MAPEG against the 4T1 cell line after 24, 48 and 72 h. Mean values (*n* = 3) and the SD (bars) are shown (x axis not in scale).

**Figure 11:**
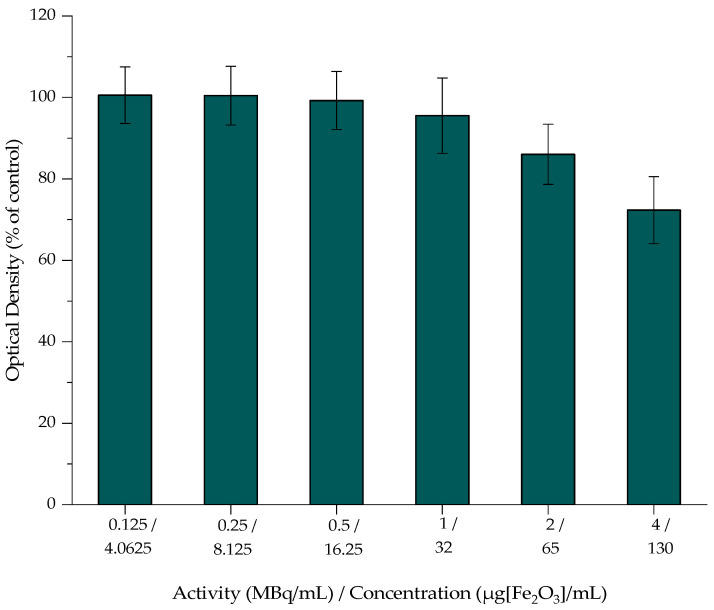
MTT assay of [^177^Lu]Lu-MAPEG against the 4T1 cell line after 24 h. Mean values (*n* = 3) and the SD (bars) are shown (x axis not in scale).

The cytotoxicity of MA, MAPEG, and [^177^Lu]Lu-MAPEG against the 4T1 breast cancer cells was investigated, and the results are presented in Figure 9, Figure 10 and Figure 11. The cytotoxic effect of the nanoparticles was evaluated after treatment with non-radiolabeled nanoconjugates up to 72 h, at concentrations starting from 130 μg/mL up to 4 μg/mL, after serial dilutions of the initial sample. Afterward, the toxicity of the ^177^Lu-labeled MAPEG was examined in the same range of concentrations used for the non-radiolabeled nanoparticles with a radioactivity range of 0.125–4 MBq/mL.

#### 3.7.1. Cytotoxicity of MA and MAPEG

In the case of MA, the viability of the cells remained high at all the studied concentrations even after 72 h of incubation (88.28 ± 3.40%). Statistical analysis was performed among the different concentrations and among the different incubation times, and all differences were non-significant.

The cytotoxicity results of MAPEG were summarized in Figure 10. They demonstrated low toxicity, even after 72 h of treatment with 130 μg[Fe_2_O_3_]/mL, with the viability remaining at almost 70%. Statistical comparison of the data produced a non-significant difference, except in the case of the two highest concentrations (65 and 130 μg[Fe_2_O_3_]/mL), as demonstrated in the following figure. Results of the 24 h experiment when compared to the corresponding concentrations at 72 h (89.81 ± 3.23% vs. 79.31 ± 8.26% and 85.94 ± 1.75% vs. 69.96 ± 6.95%) noted a significance of *p* = 0.0453 (*) and *p* = 0.0016 (**), respectively.

The MTT assay showed no remarkable toxicity effect after 3 days of treatment with either MA or MAPEG, even at the highest concentration evaluated (130 μg[Fe_2_O_3_]/mL). Previous studies by Zoppellaro et al. and by Sarigiannis et al. verified the aforementioned results for both these types of nanoparticles [42,43]. The in vitro toxicity of MagAlg against human breast adenocarcinoma cells (MCF7) resulted in ≈80% viability after 24 h. The biological impact of MagAlg-PEG followed a similar trend in the breast cancer cell line they had used (MDA-MB-231), while at the same time, they did not have the same impact on human endothelial or glioblastoma cell lines. In another study, Mag-Alg-PEG nanoparticles targeted with folic acid were evaluated with the MTT assay against MCF7 and MDA-MB-231 for 24 h and are in line with the results obtained in the present study [44].

#### 3.7.2. Cytotoxicity of [^177^Lu]Lu-MAPEG

The cytotoxic effect of the ^177^Lu-labeled MAPEG nanoparticles was evaluated in 4T1 breast cancer cells at 24 h. The radioactivity ranged between 0.125 and 4 MBq/mL, which, as summarized in Figure 11, corresponds to the non-radiolabeled MAPEG concentrations tested with MTT. At 4 MBq/mL, cell viability was 72.33 ± 8.21% versus 85.94 ± 1.75%, which corresponded to MAPEG, and their statistical differences were non-significant at all concentrations. Therefore, this difference in cell viability indicates a dose-dependent toxicity attributed to the presence of ^177^Lu.

Cell response to radiation exposure depends on the time of exposure and the activity surrounding the cells, while cytotoxicity can be implemented by the addition of a radiolabeled moiety that can actively target the cells [35]. Previous studies reported that treatment with control [^177^Lu]LuCl_3_ have shown an exponential increase in cell toxicity against RAW 264.7 cells, reaching 95% of cell growth inhibition at 120 h incubation, while at 24 h of treatment, cell viability was quite high (≈80%) [64] and similar to the results presented above. Beta emitters bound to ligands present more cell-specific toxicity and thus can be used for receptor-specific targeted endo-radiotherapy. MTT and clonogenic assays performed by Christina Müller et al. demonstrated that [^161^Tb]Tb-PSMA-617 and [^177^Lu]Lu-PSMA-617 did not affect PSMA-negative cells (PC-3 flu cells) at concentrations up to 10 MBq/mL, while the viability of PSMA-positive cells was reduced in an activity-dependent manner. [65]. Thus, MIONs decorated with ligands such as PSMA-617 could also achieve such levels of toxicity.

### 3.8. Ex Vivo Biodistribution Studies of [^68^Ga]Ga-MIONs in Normal Mice

**Figure 12:**
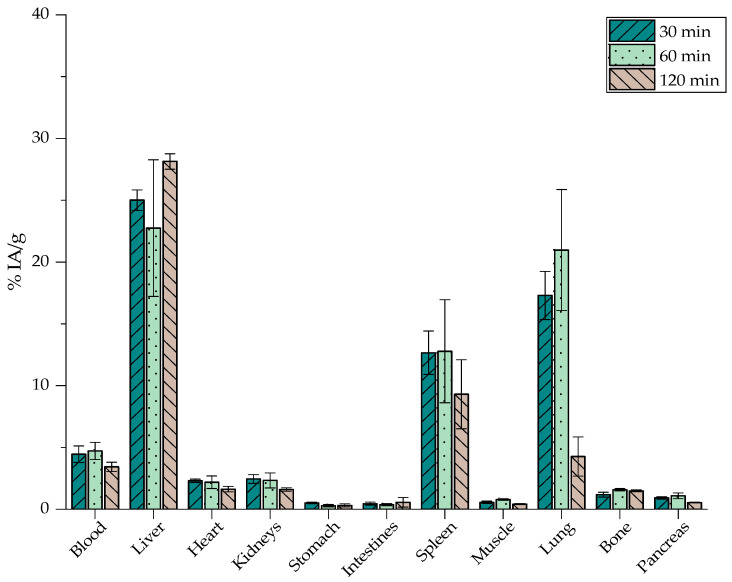
Biodistribution of [^68^Ga]Ga-MA in normal mice expressed as % IA/g (*n* = 3).

The ex vivo biodistribution studies performed in normal CFW mice were used to evaluate the in vivo kinetics of both [^68^Ga]Ga-labeled nanoparticles. [^68^Ga]Ga-MA, as shown in Figure 12, exhibit the highest accumulation in the RES organs, namely the liver at 2 h post-injection (p.i.) (28.14 ± 0.63%) and the spleen at 1 h p.i. (12.79 ± 4.17%). Furthermore, lung accumulation was noted, with a peak at 60 min p.i., reaching 20.97 ± 4.90%.

**Figure 13:**
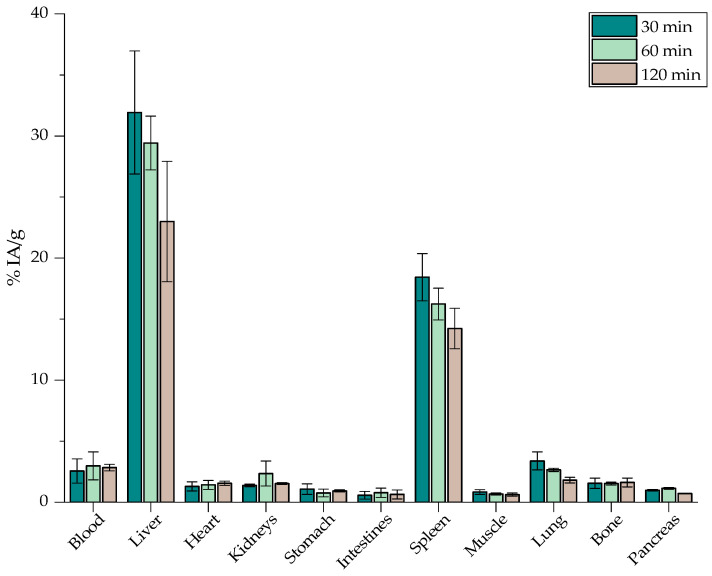
Biodistribution of [^68^Ga]Ga-MAPEG in normal mice expressed as % IA/g (*n* = 3).

The biological behavior of [^68^Ga]Ga-MAPEG is depicted in Figure 13, where an augmented liver and spleen uptake is obvious at 30 min p.i. (31.92 ± 5.04% and 18.43 ± 1.93%) followed by a decreasing pattern and reaching 22.99 ± 4.94% and 14.23 ± 1.65%, respectively, at 2 h p.i.

Figure 12 and Figure 13 demonstrate a similar in vivo behavior for the rest of the organs. Statistical analysis between [^68^Ga]Ga-MA and [^68^Ga]Ga-MAPEG demonstrated the obvious significant difference, which is observed very quickly, at 30 min in the liver, spleen, and lung uptake (25.01, 12.66 and 17.30 vs. 31.92, 18.42 and 3.38%, *p* < 0.0001). At 60 min post-injection, a statistically significant difference is observed for the liver and lungs (*p* = 0.0013 and *p* < 0.0001, respectively). Regarding [^68^Ga]Ga-MA, the highest accumulation is demonstrated in the RES organs, namely the liver at 2 h post-injection (28.14 ± 0.63%), while the pegylated counterparts show a decrease in liver uptake (22.99 ± 4.94%) at the same time point (*p* = 0.0002). This much lower tendency of the [^68^Ga]Ga-MAPEG nanoparticles to accumulate in the lungs compared to the [^68^Ga]Ga-MA observed could be attributed to the lower tendency of the pegylated particles to aggregate in vivo due to the protective PEG canopy, which provides steric stabilization, thus avoiding entrapment in lung capillaries. Low uptake of the radiolabeled nanostructures was observed in all the other major organs throughout the study.

It is well established that the fate of nanoparticles following systemic administration is highly correlated to parameters such as their size and their surface coating [66]. In general, nanoparticles of ≈ 100 nm after intravenous injection are rapidly covered by opsonins and thus recognized by cells of the MPS. Similar nanosystems reported by Papadopoulou et al., with a highly negative surface charge (−36 mV versus−40 mV in the case of [^68^Ga]Ga-MA), seem to have more interactions with macrophages and highly accumulate in organs such as the liver and spleen but also in lungs, especially at 30 min p.i. [45]. In most cases reported, surface functionalization with a biocompatible coating displays similar in vivo biodistribution as in the case of [^68^Ga]Ga-MAPEG. Gallium radiolabeled iron oxide nanoparticles described by Lahooti et al. as well as by Karageorgou et al., coated with PEG or 2,3-dicarboxypropane-1,1-diphosphonic acid (DPD), respectively, were mainly detected in the liver and spleen and remained in these organs up to 120 min p.i. [28,67].

### 3.9. Ex Vivo Biodistribution Studies of [^177^Lu]Lu-MAPEG in Normal Mice

After evaluating the biodistribution results of the [^68^Ga]Ga-MIONs and according to the 3Rs principle in animal experimentation (Replace, Reduce, Refine; Directive 2010/63/EU), only the biodistribution pattern of [^177^Lu]Lu-MAPEG was investigated due to the more favorable in vitro and ex vivo profile of the ^68^Ga-MAPEG counterpart. Since ^177^Lu is a much longer-lived isotope to be used in therapeutic applications, biodistribution experiments were conducted after 1 d, 2 d, and 7 days post-injection.

**Figure 14:**
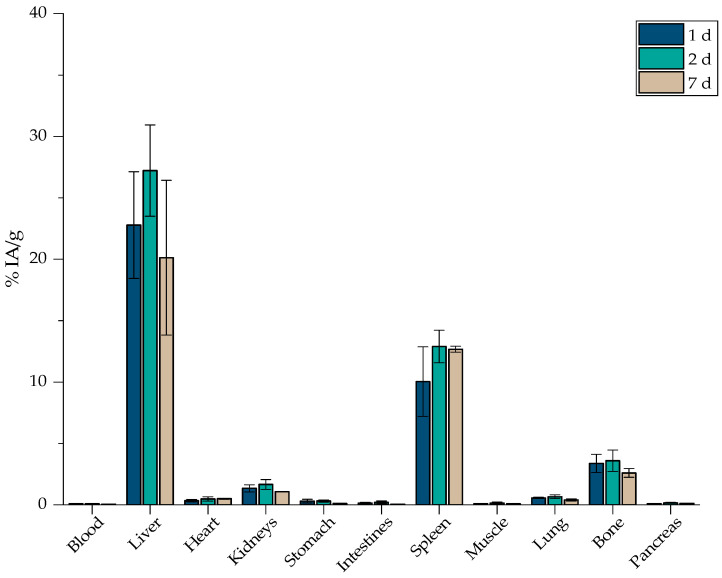
Biodistribution of [^177^Lu]Lu-MAPEG in normal mice expressed as % IA/g (*n* = 3).

The results shown in Figure 14 denote a similar behavior of [^177^Lu]Lu-MAPEG compared to [^68^Ga]Ga-MAPEG. In general, nanoparticle uptake to the liver seems to follow a slight upward trend at 1- and 2-days post-administration (22.78 ± 4.34% and 27.22 ± 3.72%, respectively) and demonstrates a stable spleen accumulation (12.89 ± 1.32% at 2 d compared to 12.67 ± 0.24% at 7 d p.i.). Nevertheless, one week after injecting the mice with [^177^Lu]Lu-MAPEG, liver uptake decreased (20.13 ± 6.29%). Liver and spleen uptake was pronounced up to 7 days p.i., while retention at the rest of the organs was below 5%. We would like to note that lung accumulation of the radiotracer follows the decreasing trend observed up to 2 h p.i in the case of [^68^Ga]Ga-MAPEG.

The ex vivo profile of the ^177^Lu-labeled MAPEG follows the rationale of a nanosystem, which with a hydrodynamic diameter of ≈ 119 nm, is mainly cleared by the hepatobiliary system and not by renal excretion as observed with nanoparticles with a hydrodynamic diameter smaller than 10 nm. Indeed, our results are in accordance with the trend of ^177^Lu-labeled SPIONs, which after 24 and 72 h p.i. showed rapid blood clearance, pronounced liver and spleen uptake, and a notably lower uptake in all other organs [38]. Gold nanoparticles radiolabeled with ^177^Lu (PEG-pGlu(^177^Lu-DOTA)_8_-LA_4_-AuNP) were evaluated by Yook et al. and demonstrated a liver and spleen accumulation of 20% at 7 d p.i., which is comparable to the results extracted from our present experiments [68].

Finally, since a high percentage of the injected dose accumulates in the liver and spleen (Figure 13 and Figure 14), MAPEG is potentially suitable for the intratumoral administration of imaging or therapeutic radionuclides rather than for systematic administration, except if the targeted tissues are the liver and spleen.

## 4. Conclusions

The MIONs investigated in the present study were first modified with alginic acid and then with poly(ethylene glycol) (PEG) in order to improve the colloidal stability and biocompatibility of the nanoparticles. Direct, fast, stable, and robust radiolabeling with the diagnostic isotope ^68^Ga and the therapeutic isotope ^177^Lu was accomplished. The hemolysis assay verified the in vitro blood compatibility of MA and MAPEG. The cytotoxicity assay showed that our conjugates did not exhibit remarkable toxicity against 4T1 cancer cells, while the ^177^Lu-labeled MAPEG indicated that viability was decreased in a dose-dependent manner. The ex vivo biodistribution of the [^68^Ga]Ga-MA in normal mice revealed high accumulation in the RES organs, whereas lung accumulation was significantly reduced in the pegylated counterparts. In the case of [^177^Lu]Lu-MAPEG, the biodistribution results demonstrated similar in vivo behavior with the ^68^Ga-radiolabeled MAPEG, with liver accumulation being the highest among all the organs.

Based on our preliminary results, we conclude that MAPEG could be further investigated as a theranostic agent, although locoregional (intratumoral) nanoparticle delivery should be the preferred route of administration to obtain significant radioactivity accumulation in tumors. To be applicable in metastatic disease, another option would be their systemic administration after their optimization by functionalization with moieties capable of targeting cancer cells.

## Data Availability

The data presented in this study are available upon request from the corresponding author P.B.
